# Both Baseline Clinical Factors and Genetic Polymorphisms Influence the Development of Severe Functional Status in Ankylosing Spondylitis

**DOI:** 10.1371/journal.pone.0043428

**Published:** 2012-09-12

**Authors:** Ruxandra Schiotis, Nerea Bartolomé, Alejandra Sánchez, Magdalena Szczypiorska, Jesús Sanz, Eduardo Cuende, Eduardo Collantes Estevez, Antonio Martínez, Diego Tejedor, Marta Artieda, Anca Buzoianu, Juan Mulero

**Affiliations:** 1 Department of Pharmacology, “Iuliu Hatieganu” University of Medicine and Pharmacy and SCBI- Rheumatology Department, Cluj-Napoca, Romania; 2 Department of Rheumatology, University Hospital “Reina Sofía”/IMIBIC, Córdoba, Spain; 3 Department of R+D, Progenika Biopharma SA, Derio-Vizcaya, Spain; 4 Department of Rheumatology, “Puerta de Hierro Majadahonda”, University Hospital, Madrid, Spain; 5 Department of Rheumatology, University Hospital “Príncipe de Asturias”, Alcalá de Henares, Madrid, Spain; 6 Department of Pharmacology, “Iuliu Hatieganu” University of Medicine and Pharmacy, Cluj-Napoca, Romania; University of Michigan, United States of America

## Abstract

Functional severity in ankylosing spondylitis (AS) patients is variable and difficult to predict early. The aim of our study was to assess whether a combination of baseline clinical factors and genetic markers may predict the development of severe functional status in AS. We performed a cross-sectional association study on AS patients included in the Spanish National Registry of Spondyloarthropathies—REGISPONSER. Bath Ankylosing Spondylitis Functional Index (BASFI) was standardized by adjusting for disease duration since the first symptoms (BASFI/t). We considered as severe functional status the values of BASFI/t in the top of the 60th (p60), 65th (p65), 70th (p70), and 75th (p75) percentile. We selected 384 single nucleotide polymorphisms (SNPs) distributed in 190 genes to be analyzed. The study cohort included 456 patients with mean age 50.8(±10.5) years and with mean disease duration since first symptoms 24.7 (±10.1) years. Older age at disease onset and neck pain at baseline showed statistical significant association with severe BASFI/t. Polymorphisms associated in the allele frequencies test with severe BASFI/t in all classifications were: *rs2542151* (p60 [*P* = .04], p65 [*P* = .04], p70 [*P* = .001] and p75 [*P* = .001]) and *rs2254441* (p60 [*P* = .004], p65 [*P* = .02], p70 [*P* = .01] and p75 [*P*<.001]).. Genotype association, after adjustment for covariates, found an association in three of the four patients' classifications for rs2542151 and in two of the classifications for rs2254441.Forward logistic regression did not identify any model with a good predictive power for severe functional development.

In our study we identified clinical factors and 24 polymorphisms associated with development of severe functional status in AS patients. Validation of these results in other cohorts is required.

## Introduction

Ankylosing spondylitis (AS) is a chronic progressive inflammatory disease affecting the spine and peripheral joints. It is largely confirmed that susceptibility to AS is genetically determined with *HLA-B27* as a major genetic contributor to the disease [1]–[3] and that environmental factors also play a role in susceptibility to the disease. In the last few years, several other genes have been reported to be involved in AS susceptibility [4]–[9].

Although the assessment of physical function is only one of several aspects of assessing disease severity, it is one of the most important measures of structural damage outcome in AS, as it directly influences the quality of life of patients and the economic costs of the disease [10]–[11]. Impairment of physical function can be subdivided into a reversible and an irreversible component. In this concept the reversible component is due to disease activity (signs and symptoms of the disease) and the irreversible component is due to structural damage that has occurred as a consequence of the disease, such as syndesmophytes and vertebral bridging. Functional severity was found to be independently determined by both the reversible factors such as disease activity and the irreversible factors such as structural damage [12] but the loss of functional capacity in each patient was not predictable from early disease stages [13].

There is evidence that several clinical parameters such as hip involvement, disease duration, erythrocyte sedimentation rate (ESR), C-reactive protein (CRP) levels, smoking, and lower socioeconomic status are associated with worse function [13]–[14]. Nevertheless, much of the variability in disease functional severity in AS remains unexplained, suggesting that genetic factors could have a greater influence than environmental factors on AS progression [13].

A genetic component has been demonstrated for AS functional severity [15]. However, very little is known about the specific genes or genetic markers inside and outside the major histocompatibility gene complex (MHC) involved in the functional component of the disease [16]–[20].

Understanding the genetic basis of functional severity in AS would be of major value to differentiate at early stages patients at high risk of severe functional impairment and patients with a lower risk. Thus, clinicians could better select and optimize the preventive and therapeutic approach for each patient as of the time of diagnosis of the disease by objectively distributing high cost treatments.

Taking into consideration the fact that impairment of physical function may be partially controlled by the appropriate treatment, the aim of our study was to identify the baseline clinical and genetic factors that determine individual development of functional severity in AS.

## Materials and Methods

### Patients with AS

We performed a cross-sectional association study on Spanish AS patients which were recruited from 25 hospitals which participated in the Spanish National Spondyloarthropathies Registry (REGISPONSER) [21]. Patients fulfilled the modified New York Criteria for AS [22] and had at least 10 years of follow-up from the first symptoms of the disease. Baseline characteristics of the patients at the beginning of the disease were recorded as potential prognostic predictors. Specifically, clinical and demographic data, sex, age at disease onset, family history of spondyloarthropathies (SpA), initial symptoms of SpA (inflammatory low back pain, neck pain, enthesitis, dactylitis, tarsitis, sacroiliac syndrome, coxitis, lower limb arthritis, and upper limb arthritis), and the number of initial SpA symptoms.

### Demographic and clinical characteristics of the AS population

The studied cohort included 456 AS patients (348 males and 108 females) with a mean age of 50.8±10.5 years, 26.1±9.1 years at disease onset and 34.6±11.4 years at diagnosis. The average time of evolution, from disease onset, was 24.7±10.1 years. *HLA-B27* was positive in 84.9% of the patients and 19.3% had a family history of spondyloarthropathies (SpA). Patients had mean BASFI at baseline 4.0±2.8, with mean BASFI/t (years) of 0.17±0.13.

### Functional phenotype

To measure functional impairment we used the BASFI score, standardized by adjusting for disease duration since first symptoms, denominated here BASFI/t. BASFI is a validated index that focuses on 10 questions pertaining to function, measured on a visual analog scale (VAS). The mean of the 10 questions generates the score, with 10 denoting worst possible functional status [23].

There are no validated threshold values for classifying AS patients into mild or severe categories according to their BASFI score standardized by disease duration (BASFI/t). Therefore, to define functional severity we performed four association analyses using the χ2-test with different criteria. Based on the opinion of the clinicians who participated in this study, who estimated that approximately 25% to 40% of AS patients had severe functional damage, we defined as severe functional status the values of BASFI/t in the top 60th (p60), 65th (p65), 70th (p70) or 75th (p75) percentiles. The cut-off values for BASFI/t severe phenotype for each percentile were: 0.19 for p60, 0.21 for p65, 0.22 for p70, and 0.25 for p75 respectively.

### HLA-B27 typing and SNP genotyping

Genomic DNA was isolated from saliva samples using the Oragene™ DNA Self-Collection kit (DNA Genotek Inc., Ottawa, Canada), according to the manufacturer's extraction protocol. All samples were tested for the presence of the HLA-B27 allele by conventional PCR using the primers reported by Olerup et al [24]. A region of 236 bp on exon 8 from the p53 gene was used as an internal control for the performance of the PCR [25].

After an extensive bibliographic search we selected 384 SNPs distributed in 190 genes to be analyzed in this study. We selected all the SNPs previously reported in Caucasians as associated with AS or with other SpA (psoriatic arthritis, juvenile idiopathic arthritis, reactive arthritis, undifferentiated arthritis, and inflammatory bowel disease–associated spondyloarthropathy). Besides those SNPs, we included some SNPs in genes described in the literature as associated to other autoimmune diseases and to bone-related disorders, since we considered them as potential candidates to be implicated in AS severity. Finally, we included tag SNPs in genes from the metabolic pathways of the two most important genes considered to be involved in AS: ERAP1 and IL-23R.The tag SNPs were selected from the HapMap CEU panel (the minor allele frequency at each locus was required to be >0.05 in Caucasian population, with an r2-value of <0.8 between adjacent markers).SNP genotyping was performed using the Illumina Golden gate genotyping platform (Illumina, Inc., San Diego, CA, USA) [26].

### Statistical analysis

Statistical analysis was performed with SPSS v 15.0 (SPSS, Chicago, IL, USA) and SVS v 7.3.1 (Golden Helix Inc., Bozeman, Montana, USA) softwares.

All quantitative data are presented as mean and standard deviation (± SD) and all qualitative data as absolute frequencies and percentages. To assess the association between clinical variables and BASFI/t severe phenotype an unvaried analysis was performed using the chi-square (χ2) test for categorical variables and the unpaired *t* test for continuous variables.

A test for deviation from Hardy-Weinberg equilibrium (HWE) was performed for each SNP. Pruning of the initial genotype dataset with default parameters (exclusion of SNPs with poor genotype cloud clustering, of SNPs with call-rate <85%, of SNPs with severe deviation from HWE (*P*<.0001) and of samples with call rate <85%) led to 456 samples and 344 SNPs [27]–[30]. Association test between allele and genotype frequencies and BASFI/t severe phenotype was performed by the chi-square (χ2) test. P-values were calculated using a single-value permutation test (1000 permutations). The minor allele frequency at all loci was above 10%…Logistic regression analysis was used to discard weather the baseline clinical factors associated with severe function could be confounding for the association between BASFI/t severe phenotype and the SNPs genotypes. P values of <.05 were considered statistically significant and p values of (.05≥p<.1) borderline.

Clinical factors and SNPs were then studied by means of multivariate logistic regression. Individual *P* values of the SNPs and of the clinical variables were ranked and only those most significantly associated with the severe functional phenotype were included in the multivariate analysis as potential predictors (*P*<.1 in the allele frequencies association test).. The multivariate analysis was performed for all four defined classifications of functional severity (p60, p65, p70, and p75). BASFI/t was considered as dependent variable and baseline clinical variables and SNPs were included as predictors. The predictive discrimination of the models was tested both by Hosmer-Lemeshow statistic and the receiver operating characteristic curve (ROC) with 95% confidence interval (CI). An area under the ROC curve (AUC) above 0.75 was considered as an indicator of a good predictive precision of the model.

### Ethical approval

This study was approved by the Ethics Committee of “Reina Sofia” University Hospital, Córdoba and “Puerta de Hierro Majadahonda” University Hospital, Madrid, Spain. Each patient signed an informed consent form upon inclusion in REGISPONSER-AS, in accordance with the fundamental principles set out in the Declaration of Human Rights in Helsinki.

## Results

### Baseline clinical variables and SNPs associated with functional severity in AS patients

Of the baseline clinical variables analyzed, the association with BASFI/t severe phenotype for neck pain and older age at disease onset was found to be statistically significant. A slight association was also found for low back pain (p60) and HLA-B27 (p65 and p70) (Table 1).

**Table 1:** Baseline clinical variables associated with functional severity in AS patients.

	p value			
Clinical Variable	BASFI/t p75	BASFI/t p70	BASFI/t p65	BASFI/t p60
**Age at disease onset**	<.001	<.001	<.001	<.001
**Neck pain**	.040	.002	.004	.011
**Low back pain**	NS	NS	NS	.030
**HLA-B27**	NS	.050	.049	NS

NS- no statistically significant.

In the allele frequencies test, from the SNPs analyzed, we identified 24 polymorphisms associated with functional severe phenotype in at least one of the patient classifications. Two SNPs showed consistent association with BASFI/t and were significantly associated in all four patient classifications: rs2542151 in the protein tyrosine phosphatase non-receptor type 2 (*PTPN2*) gene [p60 (*P* = .046), p65 (*P* = .006), p70 (*P* = .002) and p75 (*P* = .001)] and rs2254441 in the proline-serine-threonine phosphatase-interacting protein 1 (*PSTPIP1*) gene [p60 (*P* = .036), p65 (*P* = .017), p70 (*P* = .010) and p75 (*P* = .001)]. Five SNPs (*rs10065172. rs2268624, rs4986790, rs4986791, rs3736228*) were associated with BASFI/t in three of the four patient classifications and eight polymorphisms (*rs6887695, rs17481856, rs2280153, rs1217414, rs4958847, rs2227982, rs17551710, rs1248634*) in two classifications. The other nine polymorphisms (*rs6822844, rs11959820, rs3117222, rs660895, rs1061622 rs13151961, rs27044, rs6254, rs743572*) were found significantly associated with BASFI/t for only one of the patient classifications (Table 2).

**Table 2:** SNPs associated with functional severity in AS patients.

			Allele frequencies test, p value§			
SNP Name	Gene Symbol	Risk Allele	BASFI/t p75	BASFI/t p70	BASFI/t p65	BASFI/t p60
**rs2542151**	*PTPN2*	C	0.001*	0.002*	0.006*	0.046#
**rs2254441**	*PSTPIP1*	A	0.001*	0.010†	0.017#	0.036#
**rs2268624**	*TGFB3*	G	0.002*	0.017*	0,032#	NS
**rs4986790**	*TLR4*	G	0.008†	0.009†	0.037#	NS
**rs4986791**	*TLR4*	A	0.011*	0.006*	0.031#	NS
**rs10065172**	*IRGM*	G	NS	0,014#	0,012#	0.006*
**rs3736228**	*LRP5*	A	0.049#	0,051#	0,056#	NS
**rs6887695**	*IL12B*	C	NS	0.038#	0.037#	NS
**rs17481856**	*ERAP1*	G	NS	0.035#	NS	0.038#
**rs2280153**	*WISP3*	G	NS	NS	0.032#	0.014*
**rs1217414**	*PTPN22*	G	NS	0.049#	NS	0.034†
**rs4958847**	*IRGM*	G	NS	NS	0.042#	0.027#
**rs2227982**	*PDCD1*	A	NS	0.019†	0,044#	NS
**rs17551710**	*COL6A1*	A	0.043#	NS	NS	0.048#
**rs1248634**	*DLG5*	A	NS	0.047#	0.043#	NS
**rs6822844**	*IL21*	A	NS	NS	NS	0.031#
**rs11959820**	*PPARBC1B*	C	NS	NS	NS	0.017*
**rs3117222**	*LOC646702*	G	NS	NS	NS	0.018*
**rs660895**	*HLA-DRB1*	G	NS	NS	0.044†	NS
**rs1061622**	*TNFRSF1B*	A	NS	NS	NS	0.050#
**rs13151961**	*KIAA1109*	G	NS	NS	NS	0.042#
**rs27044**	*ERAP1*	C	NS	NS	NS	0.020*
**rs6254**	*PTH*	A	0,025#	NS	NS	NS
**rs743572**	*CYP17A1*	A	0.032#	NS	NS	NS

§ - The p-values shown correspond to the allele frequencies test corrected by a single-value permutation test.

The symbols represent the results of the genotype frequencies association test before and after a logistic regression analysis using the age at disease onset and neck pain as covariates:

* Significant genotype association (p<0.05) after logistic regression analysis with adjustment for age at disease onset and neck pain;

† Borderline genotype association (0.05≥p<0.1) after logistic regression analysis with adjustment for age at disease onset and neck pain;

# Significant genotype association (p<0.05) was lost after logistic regression analysis with adjustment for age at disease onset and neck pain.

As we found that age at disease onset and neck pain at onset were the clinical factors associated with BASFI/t severe phenotype, they were entered in the logistic regression modeling as covariates. The results of the genotype frequencies test showed that the SNP *rs2542151* in the *PTPN2* gene was significantly associated to BASFI/t in three of the four patients' classifications after adjustment for age at disease onset and neck pain (Table 2). There were other four SNPs with significant or borderline genotype associations in two of the patients' classifications after adjustment for age at disease onset and neck pain, *rs2254441* in the *PSTPIP1* gene, *rs2268624* in the *TGFB3* gene, and *rs4986790* and *rs4986791* in the TLR4 gene. The rest of SNPs were not associated to BASFI/t in the genotype test after correction for age at disease onset and neck pain at onset.

For further investigate the association between clinical-genetic variables and functional severe phenotype in AS, we performed a multivariate analysis using BASFI/t (severe/mild) as dependent variable and as covariates the clinical and genetic variables most significantly associated with BASFI/t ((p<0,1 in the allele frequencies association test). We did not find any model with a good predictive accuracy for BASFI/t for any of the patient classifications, p60, p65, p70, and p75 as none of the ROC curves of the predictive models attained an area under the curve (AUC) above 0.75.

## Discussion

This is a pioneering study performed on daily clinical patients which sought the clinical and genetic factors of influence in the development of functional severity in AS. Maintaining a good functional status is the main aim of pharmacologic treatment in current clinical practice. Reliable markers that could be applied early in the disease course to identify patients with potential severe functional outcome would be of major value for clinicians to consequently select the most suitable treatment strategy. The results of our study found that older age and neck pain at disease onset and several new SNPs, particularly *rs2542151* (*PTPN2*) and *rs2254441* (*PSTPIP1*), are predictors of severe functional impairment in AS. However, the combination of the genetic and clinical factors identified in our study was not sufficient to develop a predictive model with a good accuracy for AS functional outcome; therefore, additional predictors are required.

We found neck pain and older age at disease onset as the main clinical variables associated with severe AS functional status. Neck pain at onset, which in AS may arise from either mechanical or inflammatory lesions, presented a consistent association in all four patient classifications studied [p75 (*P* = .040), p70 (*P* = .002), p65 (*P* = .004), p60 (*P* = .011)]. To the best of our knowledge, this is the first study which has found neck pain as a predictor for AS functionality. There is conflicting evidence about the role of age at disease onset as a predictor of disease severity, with some studies finding an association and others failing to support this [31]–[35]. Patients included in our study were young adults at disease onset, with mean age of 26.1±9.1 years. Interestingly, we found a statistically significant association between older age at disease onset and BASFI/t severe phenotype (p75, p70, p65, p60; *P*<.001). This result is in accordance with a recent study which found that the likelihood of developing more severe radiographic damage was greater among patients with an older age at disease onset [36]. The authors suggested that this association could result if patients with an older age at disease onset are asymptomatic early in their illness or if they have occasional or milder symptoms, which might cause them to underestimate the duration of their disease. Supporting previous studies [36]–[38], we found that *HLA-B27* was poorly associated with functional severity; thus, we only identified statistically significant association in two of the percentiles studied [p65 (*P* = .050), p70 (*P* = .049)]. Surprisingly, we did not find coxitis at baseline to be associated with development of severe physical function as found in several previous studies that sought clinical predictors of functional disability in AS [39]–[41].

Recent genome-wide association studies have provided valuable evidence supporting the involvement of genetic factors in the pathogenesis and prognosis of autoimmune diseases. The two major SNPs found in this study to be associated with development of severe physical function are located in the autoinflammatory genes *PTPN2* and *PSTPIP1*. Our study found a consistent association between AS functional severity and the C allele of the SNP *rs2542151* in the *PTPN2* gene. *PTPN2* is a remarkable gene, since it appears to influence most cells involved in the development of the immune system [42]. *PTPN2* encodes the T cell protein tyrosine phosphatase TCPTP, a key negative regulator of inflammatory responses. Abnormalities in tyrosine phosphorylation have been found to be involved in the pathogenesis of numerous human diseases, such as developmental defects, neoplastic disorders, immunodeficiency, and autoimmunity [43]. The C allele of the SNP *rs2542151* at *PTPN2* gene, which we found to influence AS functional severity, was previously found to be associated with other autoimmune diseases, such as Crohn's disease, type 1 diabetes, and rheumatoid arthritis [44]–[45]. The fact that the SNP *rs2542151* in *PTPN2* is associated with different aspects of autoimmune diseases suggests that these diseases, including AS, could share common pathogenic mechanisms.

Another consistent association between poor physical function of AS patients and SNPs was found for the A allele of the polymorphism *rs2254441* in the *PSTPIP1* gene. *PSTPIP1* is a cytoskeleton-associated adaptor protein that regulates innate and adaptive immune responses. Although this pathway has been traditionally related to diseases associated with pyoderma gangraenosum, such as aseptic abscesses syndrome and chronic inflammatory bowel disease (IBD) [46], the SNP *rs2254441* has been recently reported as associated with psoriatic juvenile idiopathic arthritis, a disease included in the spondyloarthropathies group together with AS and IBD [47].

In addition to SNPs in *PTPN2* and *PSTPIP1* genes, a milder association with BASFI/t severe phenotype was observed for SNPs in the immunity-related GTPase family M protein (IRGM), transforming growth factor beta 3 (TGFB3), Toll-like receptor 4 (TLR4), and LDL receptor related protein 5 (LRP5) genes. IRGM is a human immunity-related GTPase which confers autophagic defense against intracellular pathogens such as *Mycobacterium tuberculosis* (BCG) and *Salmonella typhimurium*. This gene's polymorphism *rs10065172*, which we found associated with AS functional severity, has been associated with increased tuberculosis risk in some populations [42] and is a well-established risk factor for IBD [43].

In addition to SNPs in *PTPN2* and *PSTPIP1* genes, a milder association with BASFI/t severe phenotype was seen for SNPs in the transforming growth factor beta 3 (*TGFB3*) and Toll-like receptor 4 (*TLR4*) genes. Regarding TGFß3, high serum levels of this protein have been linked to osteoporosis risk [48] and polymorphisms in *TGFß* have been found to be associated with ossification of the posterior longitudinal ligament of the spine (OPLL) in the Japanese population [49]. To the best of our knowledge this is the first report which linked risk from allele G of *rs2268624* in *TGFß3* with development of functional severity in AS.

The association of the SNPs *rs4986790* and *rs4986791* in the *TLR4* gene could represent the involvement of the innate immune system in the progression of AS. Dysregulation of Toll-like receptor (TLR)-related pathways, specifically upregulation of TLR4 and TLR5, has been reported in AS [50].

Of particular interest was to find an association between severe functional impairment and *rs3736228* in *LRP5* gene, since a recently published study in the Chinese population has found several other SNPs in *LRP5* to be associated with AS susceptibility [47]. The SNP *rs3736228* was also identified to be associated with gender dependent bone mass formation and may be implicated in the adaptation of bone to mechanical load in humans [48]. As far as we are concerned, this is the first report in which a link between polymorphisms involved in bone formation and AS functional severity is identified.

The strength of this study lies in the large number of clinically well characterized AS patients. However, our study has some limitations. First, we performed a cross-sectional study in which we did not analyze the treatment administered to patients as a possible factor that influences physical outcome, since we did not have reliable data about which patients were on a specific therapy prior to their inclusion in the study. The lack of information about the treatment could have introduced some bias. Secondly, in spite of the clinical and genetic factors found to influence AS functional prognosis, we could not achieve a good predictive model for development of severe functional status by combining these factors. Thus, further research in this area in other cohorts or in prospective studies is needed to confirm which of these genetic markers in combination with clinical factors could identify an accurate predictive model for AS functional severity.

In conclusion, our results confirm that severe functional status in AS is associated with both clinical and genetic factors. We found that older age and neck pain at disease onset, lymphoid tyrosine phosphatase (*PTPN2*)-*rs2542151*, proline-serine-threonine phosphatase interacting protein1 (*PSTPIP1*)-*rs2254441* polymorphisms, are potential predictors of the development of severe functional status in AS patients. Validation of these results in other cohorts is required.

## References

[1] Schlosstein L, Terasaki PI, Bluestone R, Pearson CM (1973) High association of an HL-A antigen, B27, with ankylosing spondylitis. N Engl J Med 288: 704–6. 4688372 10.1056/NEJM197304052881403

[2] Brown MA, Pile KD, Kennedy LG, Campbell D, Andrew L, et al (1998) A genome-wide screen for susceptibility loci in ankylosing spondylitis. Arthritis Rheum 41: 588–95. 9550467 10.1002/1529-0131(199804)41:4<588::AID-ART5>3.0.CO;2-0

[3] Carter N, Williamson L, Kennedy LG, Brown MA, Wordsworth BP (2000) Susceptibility to ankylosing spondylitis [letter]. Rheumatology (Oxford) 39: 445. 10.1093/rheumatology/39.4.445 10817782

[4] Burton PR, Clayton DG, Cardon LR, Craddock N, Deloukas P, et al (2007) Association scan of 14,500 nonsynonymous SNPs in four diseases identifies autoimmunity variants. Nat Genet 39: 1329–37. 17952073 10.1038/ng.2007.17 PMC2680141

[5] Rueda B, Orozco G, Raya E, Fernandez-Sueiro JL, Mulero J, et al (2008) The IL23R Arg381Gln non-synonymous polymorphism confers susceptibility to ankylosing spondylitis. Ann Rheum Dis 67: 1451–4. 18199597 10.1136/ard.2007.080283

[6] Harvey D, Pointon JJ, Evans DM, Karaderi T, Farrar C, et al (2009) Investigating the genetic association between ERAP1 and ankylosing spondylitis. Hum Mol Genet 18: 4204–12. 19692350 10.1093/hmg/ddp371 PMC2758148

[7] Evans DM, Spencer CC, Pointon J, Su Z, Harvey D, et al (2011) Interaction between ERAP1 and HLA-B27 in ankylosing spondylitis implicates peptide handling in the mechanism for HLA-B27 in disease susceptibility. Nat Genet 43: 761–767. 21743469 10.1038/ng.873 PMC3640413

[8] Guo C, Xia Y, Yang Q, Qiu R, Zhao H, et al (2012) Association of the ANTXR2 gene polymorphism and ankylosing spondylitis in Chinese Han. Scand J Rheumatol 41: 29–32. 22118297 10.3109/03009742.2011.600700

[9] Lin Z, Bei JX, Shen M, Li Q, Liao Z, et al (2011) A genome-wide association study in Han Chinese identifies new susceptibility loci for ankylosing spondylitis. Nat Genet 44: 73–7. 22138694 10.1038/ng.1005

[10] Cakar E, Taskaynatan MA, Dincer U, Kiralp MZ, Durmus O, et al (2009) Work disability in ankylosing spondylitis: differences among working and work disabled patients. Clin Rheumatol 28: 1309–14. 19685294 10.1007/s10067-009-1249-1

[11] Ariza-Ariza R, Hernández-Cruz B, Collantes E, Batlle E, Fernández-Sueiro JL, et al (2009) Work disability in patients with ankylosing spondylitis. J Rheumatol 36: 2512–6. 19833749 10.3899/jrheum.090481

[12] Landewé R, Dougados M, Mielants H, van der Tempel H, van der Heijde D (2009) Physical function in ankylosing spondylitis is independently determined by both disease activity and radiographic damage of the spine. Ann Rheum Dis 68: 863–7. 18628283 10.1136/ard.2008.091793

[13] Doran MF, Brophy S, MacKay K, Taylor G, Calin A (2003) Predictors of longterm outcome in ankylosing spondylitis. J Rheumatol 30: 316–20. 12563688

[14] Cansu DU, Calışır C, Savaş Yavaş U, Kaşifoğlu T, Korkmaz C, et al (2011) Predictors of radiographic severity and functional disability in Turkish patients with ankylosing spondylitis. Clin Rheumatol 30: 557–62. 21210291 10.1007/s10067-010-1665-2

[15] Hamersma J, Cardon LR, Bradbury L, Brophy S, van der Horst-Bruinsma I, et al (2001) Is disease severity in ankylosing spondylitis genetically determined? Arthritis Rheum 44: 1396–400. 11407700 10.1002/1529-0131(200106)44:6<1396::AID-ART233>3.0.CO;2-A

[16] Brophy S, Hickey S, Menon A, Taylor G, Bradbury L, et al (2004) Concordance of disease severity among family members with ankylosing spondylitis?. J Rheumatol 31: 1775–78. 15338499

[17] Brown MA, Brophy S, Bradbury L, Hamersma J, Timms A, et al (2003) Identification of major loci controlling clinical manifestations of ankylosing spondylitis. Arthritis Rheum 48: 2234–39. 12905477 10.1002/art.11106

[18] Brown MA (2009) Progress in studies of the genetics of ankylosing spondylitis. Arthr Res & Ther 11: 254–60. 19886979 10.1186/ar2692 PMC2787301

[19] Goedecke V, Crane AM, Jaakkola E, Kaluza W, Laiho K, et al (2003) Interleukin 10 polymorphisms in ankylosing spondylitis. Genes Immun 4: 74–6. 12595905 10.1038/sj.gene.6363930

[20] Szczypiorska M, Sánchez A, Bartolomé N, Arteta D, Sanz J, et al (2011) *ERAP1* polymorphisms and haplotypes are associated with ankylosing spondylitis susceptibility and functional severity in a Spanish population. Rheumatology (Oxford) 50: 1969–75. 21865284 10.1093/rheumatology/ker229

[21] Collantes E, Zarco P, Munoz E, Juanola X, Mulero J, et al (2007) Disease pattern of spondyloarthropathies in Spain: description of the first national registry (REGISPONSER) extended report. Rheumatology (Oxford) 46: 1309–15. 17526930 10.1093/rheumatology/kem084

[22] Van der Linden S, Valkenburg HA, Cats A (1984) Evaluation of diagnostic criteria for ankylosing spondylitis. A proposal for modification of the New York criteria. Arthritis Rheum 27: 361–8. 6231933 10.1002/art.1780270401

[23] Calin A, Garrett S, Whitelock H, Kennedy LG, O'Hea J, et al (1994) A new approach to defining functional ability in ankylosing spondylitis: the development of the Bath Ankylosing Spondylitis Functional Index. J Rheumatol 21: 2281–5. 7699629

[24] Olerup O (1994) HLA-B27 typing by a group-specific PCR amplification. Tissue Antigens 43: 253–6. 8085261 10.1111/j.1399-0039.1994.tb02334.x

[25] Cho SY, Lee KG, Park SY, Lee HJ (2008) Utility of in-house PCR for HLA-B27 typing: comparison of concordance rate between PCR kit and in-house PCR. Korean J Lab Med 28: 239–43. 18594177 10.3343/kjlm.2008.28.3.239

[26] Fan JB, Oliphant A, Shen R, Kermani BG, Garcia F, et al (2003) Highly parallel SNP genotyping. Cold Spring Harb Symp Quant Biol 68: 69–78. 15338605 10.1101/sqb.2003.68.69

[27] Paynter RA, Skibola DR, Skibola CF, Buffler PA, Wiemels JL, et al (2006) Accuracy of multiplexed Illumina platform-based single-nucleotide polymorphism genotyping compared between genomic and whole genome amplified DNA collected from multiple sources. Cancer Epidemiol Biomarkers Prev 15: 2533–6. 17164381 10.1158/1055-9965.EPI-06-0219

[28] Reich D, Patterson N, De Jager PL, McDonald GJ, Waliszewska A, et al (2005) A whole-genome admixture scan finds a candidate locus for multiple sclerosis susceptibility. Nat Genet 37: 1113–8. 16186815 10.1038/ng1646

[29] Bartolomé N, Szczypiorska M, Sánchez A, Sanz J, Juanola-Roura X, et al (2012) Genetic polymorphisms, inside and outside the MHC, improve prediction of AS radiographic severity in addition to clinical variables. Rheumatology (Oxford) 51: 1471–8. 22495925 10.1093/rheumatology/kes056

[30] Low YL, Li Y, Humphreys K, Thalamuthu A, Li Y, et al (2010) Multi-variant pathway association analysis reveals the importance of genetic determinants of estrogen metabolism in breast and endometrial cancer susceptibility. PLoS Genet 6: e1001012. 20617168 10.1371/journal.pgen.1001012 PMC2895650

[31] Amor B, Santos R, Nahal R, Listrat V, Dougados M (1994) Predictive factors for the long-term outcome of spondyloarthropathies. J Rheumatol 21: 1883–7. 7837155

[32] Boonen A, vander Cruyssen B, de Vlam K, Steinfeld S, Ribbens C, et al (2009) Spinal radiographic changes in ankylosing spondylitis: association with clinical characteristics and functional outcome. J Rheumatol 36: 1249–55. 19447933 10.3899/jrheum.080831

[33] Brophy S, Calin A (2001) Ankylosing spondylitis: Interaction between genes, joints, age at onset, and disease expression. J Rheumatol 28: 2283–8. 11669170

[34] Gensler LS, Ward MM, Reveille JD, Learch TJ, Weisman MH, et al (2008) Clinical, radiographic and functional differences between juvenile-onset and adult-onset ankylosing spondylitis: results from the PSOAS cohort. Ann Rheum Dis 67: 233–7. 17604288 10.1136/ard.2007.072512

[35] Robertson LP, Davis MJ (2004) A longitudinal study of disease activity and functional status in a hospital cohort of patients with ankylosing spondylitis. Rheumatology (Oxford) 43: 1565–8. 15353608 10.1093/rheumatology/keh386

[36] Ward MM, Hendrey MR, Malley JD, Learch TJ, Davis JC Jr, et al (2009) Clinical and immunogenetic prognostic factors for radiographic severity in ankylosing spondylitis. Arthritis Rheum 61: 859–66. 19565552 10.1002/art.24585 PMC2710412

[37] Rudwaleit M, Listing J, Brandt J, Braun J, Sieper J (2004) Prediction of a major clinical response (BASDAI 50) to tumour necrosis factor a blockers in ankylosing Spondylitis. Ann Rheum Dis 63: 665–70. 15037444 10.1136/ard.2003.016386 PMC1755042

[38] Khan MA, Kushner I, Braun WE, Zachary AA, Steinberg AG (1978) *HLA-B27* homozygosity in ankylosing spondylitis: relationship to risk and severity. Tissue Antigens 11: 434–8. 694907 10.1111/j.1399-0039.1978.tb01280.x

[39] Carette S, Graham D, Little H, Rubenstein J, Rosen P (1983) The natural disease course of ankylosing spondylitis. Arthritis Rheum 26: 186–90. 6600615 10.1002/art.1780260210

[40] Falkenbach A, Franke A, van der Linden S (2003) Factors associated with body function and disability in patients with ankylosing spondylitis: a cross-sectional study. J Rheumatol 30: 2186–92. 14528516

[41] Vander Cruyssen B, Muñoz-Gomariz E, Font P, Mulero J, de Vlam K (2010) al. ASPECT-REGISPONSER-RESPONDIA working group (2010) Hip involvement in ankylosing spondylitis: epidemiology and risk factors associated with hip replacement surgery. Rheumatology (Oxford) 49: 73–81. 19605374 10.1093/rheumatology/kep174

[42] Moore F, Colli ML, Cnop M, Esteve MI, Cardozo AK, et al (2009) PTPN2, a candidate gene for type 1 diabetes, modulates interferon-gamma-induced pancreatic beta-cell apoptosis. Diabetes 58: 1283–91. 19336676 10.2337/db08-1510 PMC2682688

[43] Vang T, Miletic AV, Arimura Y, Tautz L, Rickert RC, et al (2008) Protein tyrosine phosphatases in autoimmunity. Annu Rev Immunol 26: 29–55. 18303998 10.1146/annurev.immunol.26.021607.090418

[44] Burton PR, Clayton DG, Cardon LR, Craddock N, Deloukas P, et al (2007) Genome-wide association study of 14,000 cases of seven common diseases and 3,000 shared controls. Nature 447: 661–78. 17554300 10.1038/nature05911 PMC2719288

[45] Morgan AR, Han DY, Huebner C, Lam WJ, Fraser AG, et al (2010) *PTPN2* but not *PTPN22* is associated with Crohn's disease in a New Zealand population. Tissue Antigens 76: 119–25. 20403149 10.1111/j.1399-0039.2010.01493.x

[46] Wollina U, Haroske G (2011) Pyoderma gangraenosum. Curr Opin Rheumatol 23: 50–6. 21037478 10.1097/BOR.0b013e328341152f

[47] Day TG, Ramanan AV, Hinks A, Lamb R, Packham J, et al (2008) Autoinflammatory Genes and Susceptibility to Psoriatic Juvenile Idiopathic Arthritis. Arthritis Rheum 58: 2142–6. 18576390 10.1002/art.23604 PMC2688675

[48] Grainger DJ, Percival J, Chiano M, Spector TD (1999) The role of serum TGF-beta isoforms as potential markers of osteoporosis. Osteoporos Int 9: 398–404. 10550458 10.1007/s001980050163

[49] Kamiya M, Harada A, Mizuno M, Iwata H, Yamada Y (2001) Association between a polymorphism of the transforming growth factor-beta1 gene and genetic susceptibility to ossification of the posterior longitudinal ligament in Japanese patients. Spine 26: 1264–1267. 11389394 10.1097/00007632-200106010-00017

[50] Assassi S, Reveille JD, Arnett FC, Weisman MH, Ward MM, et al (2010) Whole-blood gene expression profiling in Ankylosing Spondylitis shows upregulation of Toll-like receptor 4 and 5. J Rheumatol 38: 87–98. 20952467 10.3899/jrheum.100469 PMC3014385

